# Accuracy of on-site teleoperated milling with haptic assistance

**DOI:** 10.1007/s11548-023-02983-2

**Published:** 2023-07-16

**Authors:** Sergey Drobinsky, Matías de la Fuente, Behrus Puladi, Klaus Radermacher

**Affiliations:** 1 Chair of Medical Engineering, RWTH Aachen University, Aachen, Germany; 2 Department of Oral and Maxillofacial Surgery, University Hospital RWTH Aachen, Aachen, Germany; 3 Institute of Medical Informatics, University Hospital RWTH Aachen, Aachen, Germany

**Keywords:** Cooperative robotic assistants, Virtual fixtures, Haptic assistance

## Abstract

**Purpose:**

In bone surgery specialties, like orthopedics, neurosurgery, and oral and maxillofacial surgery patient safety and treatment success depends on the accurate implementation of computer-based surgical plans. Unintentional plan deviations can result in long-term functional damage to the patient. With on-site teleoperation, the surgeon operates a slave robot with a physically-decoupled master device, while being directly present at the operation site. This allows the surgeon to perform surgical tasks with robotic accuracy, while always remaining in the control loop.

**Methods:**

In this study the master- and slave-side accuracy of an on-site teleoperated miniature cooperative robot (minaroHD) is evaluated. Master-side accuracy is investigated in a user study regarding scale factor, target feed rate, movement direction and haptic guidance stiffness. Scale factors are chosen to correspond to primarily finger, hand, and arm movements. Slave-side accuracy is investigated in autonomous milling trials regarding stepover, feed rate, movement direction, and material density.

**Results:**

Master-side user input errors increase with increasing target feed rate and scale factor, and decrease with increasing haptic guidance stiffness. Resulting slave-side errors decrease with increasing scale factor and are < 0.07 mm for optimal guidance parameters. Slave-side robot position errors correlate with the feed rate but show little correlation with stepover distance. For optimal milling parameters, the 95th percentile of tracked slave-side position error is 0.086 mm with a maximal error of 0.16 mm.

**Conclusion:**

For optimal guidance and milling parameters, the combined error of 0.23 mm is in the range of the dura mater thickness (< 0.27 mm) or mandibular canal wall (~ 0.85 mm). This corresponds to safety margins in high-demand surgical procedures like craniotomies, laminectomies, or decortication of the jaw. However, for further clinical translation, the performance and usability of on-site teleoperated milling must be further evaluated for real-life clinical application examples with consideration of all error sources in a computer-assisted surgery workflow.

## Introduction

Accurate plan execution is crucial for patient safety and successful treatment outcomes in surgical specialties which involve bone-related procedures, such as orthopedic surgery, neurosurgery, and oral and maxillofacial surgery. To execute plans accurately, surgeons must perform complex, temporo-spatial mental transformations. The necessary expert-level technical skills require sustained and intentional practice [1]. Errors, such as inaccurate determination of anatomical landmark and misjudging distances can have considerable consequences [2]. For milling tasks both, exceeding pre-planned boundaries and not removing enough bone can have serious implications for patient safety and treatment success. Exceeding boundaries can cause implant misalignment, functional restrictions, and damage to sensitive tissues, such as vessels and nerves. Not removing enough bone can lead to poor implant fit and recurrence of bone tumors. Thereby, novice surgeons may have limited opportunities to acquire the necessary experience for mastering complex treatments, especially in non-specialized hospitals and for low-volume treatments.

By providing haptic assistance, robots can augment surgeon’s sensorimotor skills (planning-independent assistance) and support accurate plan implementation (patient-specific planning-dependent assistance) [3]. However, safe deployment of autonomous robots, like in industrial applications, is challenging in the unstructured and dynamic clinical work environment. Cooperative robots offer a promising alternative by combining the accuracy of the robot with the expert decision-making abilities of the surgeon [4, 5]. Previous research already demonstrated benefits of haptic assistance for pose finding, trajectory following and volumetrically constrained surgical tasks [3, 6–8]. Thereby, adequate design of the human–machine interaction contributes significantly to reducing preventable errors when introducing new technologies in complex socio-technical surgical systems [9, 10]. Furthermore, miniaturization and modularization of surgical robots are additional key challenges for safe, versatile, and cost-effective robot deployment [8, 11].

Regarding human–robot interaction, current cooperative surgical robots can be classified into handheld, hands-on, and teleoperated systems [12]. The range of possible cooperative functions and consequently the scope of application depends on the system type [3]. Hand-held systems allow surgeons to pre-position, re-position, and retract the robot at any time, but do not allow for interactive adjustment of the executed motion. Hand-held robots essentially act autonomously within their range of motion. Hands-on systems allow direct interaction with the tool and the robot, providing haptic assistance and direct haptic feedback from the patient's anatomy. However, motion and force transformation are not possible. Teleoperated systems, on the other hand, physically decouple the surgeon from the surgical instrument, allowing flexible motion and force transformation, such as scaling. However, due to this physical decoupling, haptic feedback from the patient is inherently unavailable. If necessary, haptic feedback can be restored using force/torque sensors and haptic input devices. Additionally, virtual guidance forces can be generated to provide haptic assistance. On-site teleoperation allows surgeons to return to the bedside and regain a direct view of the patient, while retaining all the benefits of teleoperation assistance [8].

The minaroHD is a miniature milling robot that can be used in handheld, hands-on and teleoperated configurations, while autonomously compensating for patient movements of 18 mm/s with an accuracy of 0.5 mm [13]. The aim of this study is to investigate the effect of milling and haptic guidance parameters on the milling accuracy, specifically regarding master-side input errors and slave-side positioning errors for on-site teleoperated milling with haptic assistance.

## Material and methods

**Fig. 1:**
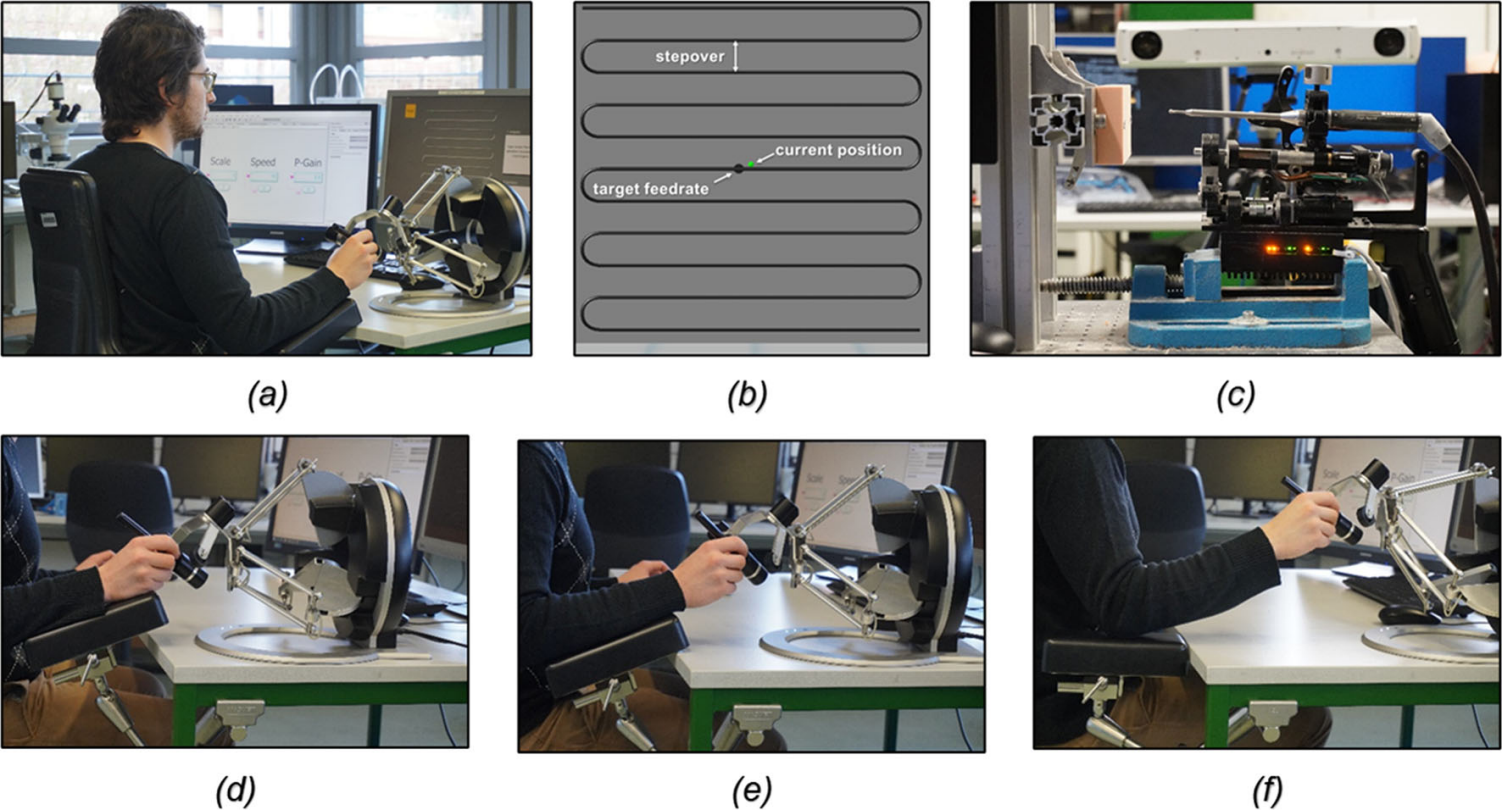
Master-side test setup for teleoperated path tracing (**a**) and the traced trajectory (**b**) with different arm rest adjustments: wrist support for primary finger movements (scale factor 1) (**d**), forearm support for primary hand movements (scale factor 2,5) (**e**), and elbow support for free lower arm movements (scale factor 5) (**f**). Slave-side test setup for the autonomous milling trials with the minaroHD (**c**)

This study investigated the achievable accuracy of teleoperated milling with haptic assistance. Two error sources are considered: user inputs on the master side and the accuracy of the respective robot movement on the slave side (Fig. 1a, c). In test case 1, the accuracy of the master-side user input is evaluated for tracing paths with haptic assistance. The study was conducted with non-expert users to investigate the achievable fine motor performance with haptic assistance regardless of technical expertise. In test case 2, the slave-side position accuracy of the robot is investigated in autonomous milling trials with generated ideal user inputs. Descriptive statistical data analysis was performed using Matlab (2018b, Mathworks, Natick, MA, USA).

In test case 1, subjects were asked to trace a path using the Omega.6 haptic device (ForceDimension, Nyon, Switzerland) while receiving haptic assistance. Therefore, the path-tracing accuracy of the subjects on the master side was investigated with respect to master-side scale factor ($sf$ = 1, 2.5, 5), slave-side target feed rate (${v}_{\mathrm{feed}}$ = 5 mm/s, 15 mm/s), movement direction (horizontal, vertical), and haptic guidance stiffness ($p$ = 0.5, 0.25 N/mm, $d$ = 0.03 Ns/mm) in a full-factorial design (24 test runs). Scale factors were chosen to be appropriate for finger, hand, and arm movements, respectively. The arm rest was individually adjusted to provide comfortable wrist support for primary finger movements ($sf$ = 1), forearm support for primary hand movements ($sf$ = 2.5), and elbow support for free arm movements ($sf$ = 5) (Fig. 1d–f). Due to motion scaling, the actual required master-side tracing speed was the product of master-side scale factor and slave-side target feed rate. Haptic assistance and a visual representation of the virtual environment were implemented using Matlab Simulink (2018b, Mathworks, Natick, MA, USA) and the real-time add-on Quarc (2019 SP1, Quanser, Markham, Canada) as described in [7].

First, subjects were seated in front of a monitor and the haptic device was positioned in front of the subject’s dominant hand. Prior to the experiments, subjects were given an interactive tutorial to familiarize themselves with the system and the provided haptic and visual assistance. After the tutorial was completed, the subjects were asked to trace a two-dimensional zig-zag milling path with 11 straight segments sized 20 × 20 mm (stepover 2 mm) within each test run (Fig. 1b). Haptic assistance was provided in all three spatial dimensions, while visual feedback was only provided in two dimensions parallel to the traced trajectory (main-visual plane). The resulting deviations on the slave-side were visually displayed in a virtual environment with a magnification factor 11.6 on a G2200WT Monitor (Benq, Taipei, Taiwan) (pixel distance 0.29 mm) (Fig. 1b). Subjects completed 24 test runs in a random sequence grouped by scale, to reduce the number of necessary arm rest adjustments. Prior to each test run, subjects were informed about the tested parameter configuration and were shown a visual reference for the target feed rate. Subjects were asked to prioritize tracing accuracy and to consider the target feed rate as a secondary goal. Path deviations were assessed on the straight segments of the trajectory, excluding the first segment to account for an adjustment period to reach the target feed rate.

In test case 2, the slave robot’s positioning error was investigated in autonomous milling trials with respect to the milling parameters feed rate (${v}_{\mathrm{feed}}$ = 5–30 mm/s in 5 mm/s steps), stepover ($x$ = 0.2–2 mm in 0.2 mm steps), direction of burr rotation (upcut, downcut), direction of movement (vertical and horizontal) and material density ($\varrho$ = 240 kg/m^3^, 1200 kg/m3). A constant stepover of $x$ = 2 mm was used for variable feed rate trials. A constant feed rate of ${v}_{\mathrm{feed}}$ = 15 mm/s was used for variable stepover trials. Ideal master-side user inputs without path deviations were generated and sent via a TCP/IP connection to the robot controller on the slave side. An Anspach handpiece (JNJ, New Brunswick, NJ, USA) with a 4 mm ball burr was mounted on the minaroHD miniature robot [13] and calibrated with an optically tracked calibration plate. For milling, low-density blocks made of SikaBlock M330 (Sika Deutschland GmbH, Bad Urach, Germany) and high-density blocks made of Obomodulan 1200 sahara (OBO-Werke GmbH, Stadthagen, Germany) were used. The blocks were mounted on a metal frame and registered using the iterative closest point algorithm. The robot and the milling block mount were tracked during milling trials with a fusionTrack 500 stereo camera (Atracsysx, Puidoux, Switzerland) (update rate 335 Hz, 0.09 mm RMS) and passive marker spheres.

## Results

### Test case 1: haptically-assisted path tracing

Nine subjects from academia (Chair of Medical Engineering) without surgical background and without prior experience with the system (7 male, 2 female, 8 right-handed, 1 left-handed, 20–50 y/o) participated in the study. Four subjects had to repeat failed test runs (${v}_{\mathrm{feed}}$ = 15 mm/s, $p$ = 0.5 N/mm, $sf$ = 1, 2.5), because the haptic guidance reference point (proxy) was locked due to excessive path deviations, preventing subjects from completing the test run. One subjects could not complete a test run (${v}_{\mathrm{feed}}$ = 15 mm/s, $p$ = 0.5 N/mm, $sf$ = 2.5) despite multiple attempts. The subjects’ average tracing speed was 5.2 ± 2.2 mm/s for test runs with target feed rate ${v}_{\mathrm{feed}}$ = 5 mm/s and 16.27 ± 5.86 mm/s for test runs with target feed rate ${v}_{\mathrm{feed}}$ = 15 mm/s. Mean path deviations averaged over all subjects show no correlation with the chronological order in which test runs were completed by the user, indicating that learning or fatigue effects are negligible (Pearson’s *r* = 0.13).

**Fig. 2:**
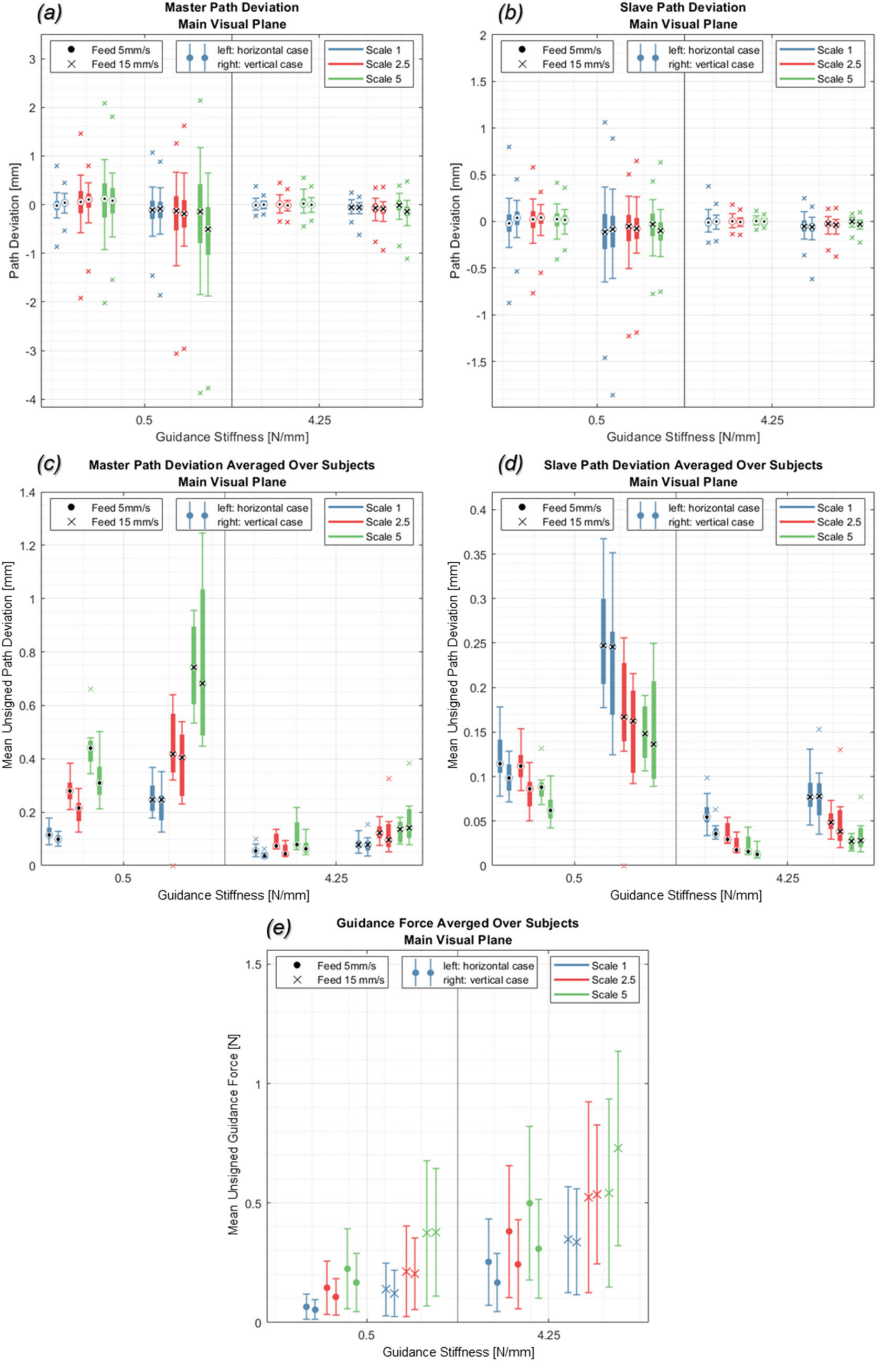
Signed mean and standard deviation of path deviation on master (**a**) and slave (**b**) side and unsigned mean path deviation for master (**c**) and slave side (**d**) (axes for master and slave side are scaled differently for better visibility). Signed mean and standard deviation of provided guidance force (**e**)

Signed path deviations show how far subjects deviated from the path on the master side and the resulting deviations that would occur on the slave side (Fig. 2a, b, marker: median, box: 25th/75th, whiskers: 5th/95th percentile). On the slave side, the 95th percentile deviations are within ± 0.7 mm (max. deviations ± 1.9 mm) for low-stiffness guidance ($p\hspace{0.17em}$ = 0.5 N/mm), and within ± 0.4 mm (max. deviations ± 0.7 mm) for high-stiffness guidance ($p\hspace{0.17em}$ = 4.25 N/mm). For the test run with the highest achieved tracing accuracy ($p\hspace{0.17em}$ = 4.25 N/mm, ${v}_{\mathrm{feed}}\hspace{0.17em}$ = 5 mm/s, $sf$ = 5) the 95th percentile deviation is within ± 0.03 mm (max. deviations ± 0.07 mm). On the master side, the 95th percentile deviations are within ± 1.9 mm (max. deviations ± 3.87 mm), while for the test run with the highest tracing accuracy ($p\hspace{0.17em}$ = 4.25 N/mm, ${v}_{\mathrm{feed}}$ = 5 mm/s, $sf$ = 1) the 95th percentile deviation is within ± 0.09 mm (max. deviation ± 0.2 mm).

The mean unsigned path deviations show the relationship between tracing accuracy and guidance parameters (Fig. 2 c-d, marker: median, box: 25th/75th, outliers: > 1.5 × interquartile range). With increasing scale factors, position errors decrease on the slave side but increase on the master side. On both sides, master and slave side, position errors increase with rising feed rates and decrease with rising guidance stiffness. For high-stiffness guidance, scale and feed rate have a small effect on the position error ($\Delta {p}_{\mathrm{err},\mathrm{master}}$ = 0.1 mm, $\Delta {p}_{\mathrm{err},\mathrm{slave}}$ = 0.07 mm) compared to low-stiffness guidance ($\Delta {p}_{\mathrm{err},\mathrm{master}}$ = 0.64 mm, $\Delta {p}_{\mathrm{err},\mathrm{slave}}$ = 0.18 mm).

Guidance forces show the amount of haptic assistance subjects received (Fig. 2e, marker: mean, errorbar: standard deviation). In the main visual plane, highest guidance forces were 0.73 ± 0.4 N ($p$ = 4.25 N/mm, ${v}_{\mathrm{feed}}$ = 15 mm/s and $sf$ = 5) and lowest guidance forces were 0.05 ± 0.04 N ($p$ = 0.5 N/mm, ${v}_{\mathrm{feed}}$ = 5 mm/s and $sf$ = 1). Guidance forces increase with the scale factor, feed rate and guidance stiffness. In the depth direction, where no visual feedback was provided, guidance forces are higher and independent of test run parameters ($F$ = 1.15 ± 0.57 N). For the trial with the lowest guidance forces, the 95th percentile of signed path deviations on the slave side was within ± 0.28 mm (max. deviations ± 0.87 mm).

### Test case 2: autonomous milling

**Fig. 3:**
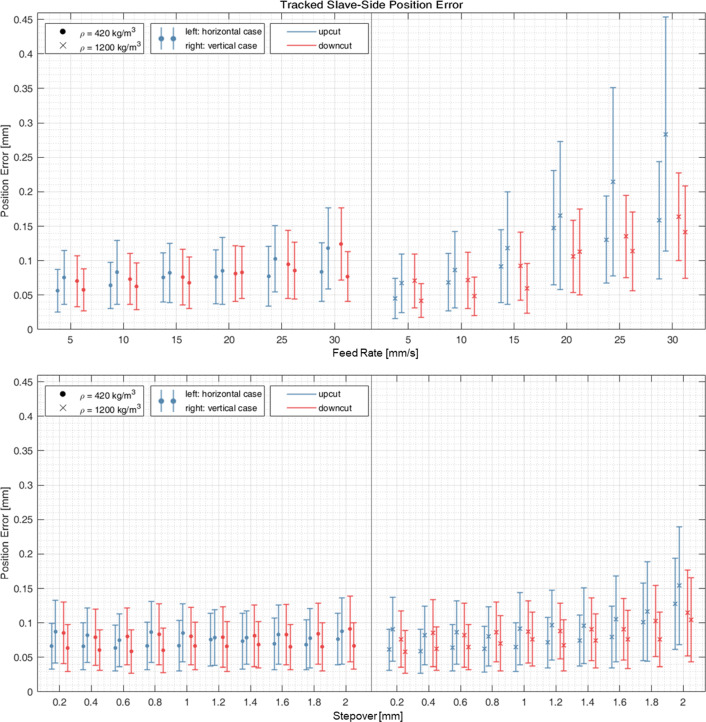
Tracked slave-side error (mean and standard deviation) for teleoperated minaroHD with generated optimal user inputs for variable feed rate (stepover $x$ = 2 mm) and variable stepover (feed rate ${v}_{\mathrm{feed}}$ = 15 mm/s)

Tracked position errors of the slave robot (unsigned path deviations) for generated optimal user inputs on the master side are shown in Fig. 3 (marker: mean error, errorbar: standard deviation). For path deviation and stepover, no correlation (Pearson’s *r* < 0.1) was found for milling in low-density material ($\varrho$ = 240 kg/m^3^), and weak positive correlation (*r* < 0.31) was found for milling in high-density material ($\varrho$=1200 kg/m^3^). For variable stepover trials, mean path deviations are below 0.09 mm for milling low-density material and 0.15 mm for high-density material. For path deviation and feed rate, weak positive correlation was found for milling in low-density material, and strong positive correlation (*r* > 0.5) for milling in high-density material. For variable feed rate trials, mean path deviations are below 0.12 mm for milling low-density material and 0.28 mm for high-density material. The lower-bound of tracked mean path deviations is 0.04 mm (${v}_{\mathrm{feed}}$=5 mm/s, $x$=2 mm $\varrho$=1200 kg/m^3^). For trials with the highest deviations (${v}_{\mathrm{feed}}$=30 mm/s, $x$=2 mm $\varrho$=1200 kg/m^3^), the 95th percentile error was 0.61 mm (maximal error 0.8 mm) and for trials with the lowest deviations (${v}_{\mathrm{feed}}$=5 mm/s, $x$=2 mm, $\varrho$=1200 kg/m^3^) 95th percentile error was 0.086 mm (maximal error 0.16 mm). In static trials, where the robot was commanded to hold a position for 15 s, the maximal position error measured by the robot’s internal encoders was 0.0058 mm.

## Discussion

The achieved tracing accuracy on the master side is in line with the results of Schleer et al. [7], where mean unsigned path deviations of 0.3 mm with and 0.5 mm without haptic assistance were achieved (${v}_{\mathrm{feed}}$=15 mm/s). This study shows that higher tracing accuracies of up to 0.07 mm on the slave side can be achieved with higher stiffness guidance or motion scaling. While, high guidance forces prevent unintentional path deviations, they also limit the surgeon’s ability to intentionally deviate from the path when necessary. With less restrictive haptic assistance configurations (low stiffness, no scaling) but in combination with low tracing speeds and a magnified view, tracing accuracies of up to ± 0.28 mm (95th percentile) are possible.

The observed lower bound of required guidance forces 0.05 N is in line with the hand’s perception threshold reported in literature [14]. Actual provided guidance forces may vary due to calibration errors of the haptic device. The observed increase of guidance forces with increasing scale factors may be due to the increased inertia or lower force perception sensitivity of finger, hand, and arm movements, respectively. Alternatively, as the slave-side path deviations, which were displayed in the virtual environment, decreased with increasing scale factors, the reduced visual feedback may also be a contributing factor for an increased demand for haptic assistance.

With generated ideal user inputs at the master side, mean tracked position error of the minaroHD on the slave side were below 0.12 mm for milling in low-density material ($\varrho$=240 kg/m^3^) and 0.28 for milling high-density material ($\varrho$=1200 kg/m^3^). Observed tracked position errors at different feed rates are comparable to the motion compensation accuracy reported by Vossel et al. [13]. This indicates that, compared to the control error, milling forces have no apparent effect on the position error for the presented test-setup and the investigated range of milling parameters. However, the tracked position error does not consider tracking errors and deformations of the burr relative to the tracking array. Lower bounds of mean tracked position errors (0.04 mm) are therefore of limited reliability, as they are below the measurement precision of the used tracking camera (0.09 mm).

In conclusion, for best case haptic guidance and milling configurations a combined accuracy resulting from user input errors (< 0.07 mm) and slave position errors (< 0.16 mm) of 0.23 mm is theoretically achievable on the slave side for on-site teleoperated milling with the minaroHD robot. However, several error sources that would occur in real clinical applications were not considered in this study: On the master side, the accuracy of the provided haptic and visual guidance depends on the preoperative segmentation accuracy and the intraoperative plan registration accuracy. For image-based navigation, modern algorithms achieve average surface distances of up to 0.31 mm for segmentation of bone from computer tomography images [15]. For manual bone registration with anatomical landmarks registration errors of 1 mm and 1.2° can occur [16]. On the slave side, the accuracy of the robot also depends on the tracking accuracy and the rigidity of the burr and the burr mount. For clinical applications, compensation of patient movement would introduce additional control errors, while the elasticity of an appropriate robot mount would limit the speed at which stable motion compensation is possible. Finally, when subjects rely on the direct view of the situs rather than a simulated navigation display, latency between master input and slave motion may introduce additional errors.

Safety margins for orthopedic and neurosurgical procedures like laminectomies and craniotomies may be in the range of the dura mater thickness (0.27–0.35 mm) [17]. In oral and maxillofacial procedures like decortication of the jaw safety margins can be in the range of the wall thickness of the mandibular nerve canal (0.86 ± 0.18 mm) [18]. Therefore, further investigations are necessary to prove that the proposed approach can satisfy the accuracy requirements for high-demand surgical applications in orthopedics, neurosurgery, and oral and maxillofacial surgery.

## Conclusion

Cooperative robotic assistance can potentially help surgeons to successfully perform increasingly demanding surgical procedures by augmenting the sensorimotor abilities and supporting accurate implementation of surgical plans. On-site teleoperation creates thereby new possibilities to combine the wide variety of assistance functions possible with teleoperation (e.g., motion scaling or tremor filtering) and the ability to directly see and interact with the patient when necessary. The presented study shows that the investigated milling and guidance parameters have a considerable effect on the resulting milling errors and therefore need to be carefully chosen. Thereby, haptic guidance parameters need to satisfy task requirements not only in terms of required accuracy, but also range of motion and the desired guidance compliance. Excessively restrictive haptic assistance can limit the surgeon’s agency and lead to automation errors like overreliance or reduced situational awareness [19, 20]. For clinical translation, the usability and performance of the presented on-site teleoperated approach must be further investigated for real-life clinical application examples with consideration of all errors sources in a computer assisted surgery workflow.

## Data Availability

This article does not contain patient data. Data from lab study participants are anonymized.
